# Confirmation of Cause of Death Via Comprehensive Autopsy and Whole Exome Molecular Sequencing in People With Epilepsy and Sudden Unexpected Death

**DOI:** 10.1161/JAHA.121.021170

**Published:** 2021-11-24

**Authors:** C. Anwar A. Chahal, David J. Tester, Ahmed U. Fayyaz, Keerthi Jaliparthy, Nadeem A. Khan, Dongmei Lu, Mariha Khan, Aradhana Sahoo, Aiswarya Rajendran, Jennifer A. Knight, Michael A. Simpson, Elijah R. Behr, Elson L. So, Erik K. St. Louis, R. Ross Reichard, William D. Edwards, Michael J. Ackerman, Virend K. Somers

**Affiliations:** ^1^ Mayo Clinic Graduate School of Biomedical Sciences Mayo Clinic Rochester MN; ^2^ WellSpan Center for Inherited Cardiovascular Diseases WellSpan Health PA; ^3^ Department of Cardiovascular Medicine Mayo Clinic Rochester MN; ^4^ Division of Cardiology Department of Medicine University of Pennsylvania Philadelphia PA; ^5^ Department of Molecular Pharmacology & Experimental Therapeutics Windland Smith Rice Sudden Death Genomic Laboratory Mayo Clinic Rochester MN; ^6^ Department of Laboratory Medicine & Pathology Mayo Clinic Rochester MN; ^7^ Department of Medicine Mayo Clinic Rochester MN; ^8^ Mayo Clinic College of Medicine Mayo Clinic Rochester MN; ^9^ King’s College London London United Kingdom; ^10^ Cardiology Section and Cardiovascular Clinical Academic Group St George’s, University of London London United Kingdom; ^11^ St George’s University Hospitals’ NHS Foundation Trust London United Kingdom; ^12^ Department of Neurology Mayo Clinic Rochester MN; ^13^ Mayo Center for Sleep Medicine Mayo Clinic Rochester MN; ^14^ Department of Pediatrics Mayo Clinic Rochester MN

**Keywords:** cardiomyopathies, channelopathies, genetics, sudden death, Cardiomyopathy, Epidemiology, Genetics, Ion Channels/Membrane Transport, Sudden Cardiac Death

## Abstract

**Background:**

Sudden cardiac arrest is the leading mode of death in the United States. Epilepsy affects 1% of Americans; yet epidemiological data show a prevalence of 4% in cases of sudden cardiac arrest. Sudden unexpected death in epilepsy (SUDEP) may share features with sudden cardiac arrest. The objective of this study was to report autopsy and genomic findings in a large cohort of SUDEP cases.

**Methods and Results:**

Mayo Clinic Sudden Death Registry containing cases (ages 0–90 years) of sudden unexpected and unexplained deaths 1960 to present was queried. Exome sequencing performed on decedent cases. From 13 687 cases of sudden death, 656 (4.8%) had a history of seizures, including 368 confirmed by electroencephalography, 96 classified as SUDEP, 58 as non‐SUDEP, and 214 as unknown (insufficient records). Mean age of death in SUDEP was 37 (±19.7) years; 56 (58.3%) were male; 65% of deaths occurred at night; 54% were found in bed; and 80.6% were prone. Autopsies were obtained in 83 cases; bystander coronary artery disease was frequently reported as cause of death; nonspecific fibrosis was seen in 32.6% of cases, in structurally normal hearts. There were 4 cases of Dravet syndrome with pathogenic variants in *SCN1A* gene. Using whole exome sequencing in 11 cases, 18 ultrarare nonsynonymous variants were identified in 6 cases including *CACNB2, RYR2, CLNB, CACNA1H,* and *CLCN2*.

**Conclusions:**

This study examined one of the largest single‐center US series of SUDEP cases. Several cases were reclassified as SUDEP, 15% had an ECG when alive, and 11 (11.4%) had blood for whole exome sequencing analysis. The most frequent antemortem genetic finding was pathogenic variants in *SCN1A*; postmortem whole exome sequencing identified 18 ultrarare variants.

**Nonstandard Abbreviations and Acronyms**
DrS: Dravet syndrome
PWE: people with epilepsy
SCD: sudden cardiac death
SUDEP: sudden unexpected death in epilepsy

**Clinical Perspective**

**What Is New?**

- Sudden unexpected death in epilepsy is still not well understood and it can overlap with sudden cardiac arrest in many cases.
- We collected one of the largest cohorts of sudden unexpected death in epilepsy cases with the aim to perform postmortem autopsy and antemortem‐postmortem genetic testing with any available DNA.
- The most frequent pathogenic variants reported were in the *SCN1A* gene related to Dravet syndrome and we found 18 ultrarare variants of undetermined significance in 11 cases.

**What Are the Clinical Implications?**

- Our findings suggest a serious gap in knowledge of the genetic associations of sudden unexpected death in epilepsy because of the lack of sufficient blood and tissue samples to be analyzed and the urgent need for a large‐scale, multicenter, cooperative prospective sudden death registry in people with epilepsy to yield analyzable samples and to elucidate possible causative variants.

Sudden cardiac arrest is the leading cause of death in the United States, claiming 340 000 lives each year, including thousands under the age of 35 who tragically die suddenly and unexpectedly.^1^, ^2^, ^3^ Epilepsy affects 1% of Americans, yet epidemiological data of victims of sudden cardiac arrest show a prevalence of preexisting epilepsy of 4%, suggesting these cases of sudden cardiac arrest could instead be sudden unexpected death in epilepsy (SUDEP).^1^, ^2^, ^3^ More than two‐thirds of witnessed arrests in people with epilepsy (PWE) have no preceding seizure activity^4^, ^5^, ^6^ and a disproportionate 60% of SUDEP fatalities occur during sleep, implicating cardiac arrhythmia and possible undiagnosed sleep apnea as possible contributors.^7^, ^8^, ^9^, ^10^, ^11^

SUDEP can occur at any age, including infants and children, but those between the ages of 15 to 40 years are at greatest risk. The young age of victims makes SUDEP a highly visible, emotive, and tragic event, and in potential years of life lost it is the leading cause of death after stroke.^12^, ^13^, ^14^, ^15^

Although the pathophysiology of SUDEP is unsolved, postictal brain shutdown with electroencephalography suppression, depression of respiratory function, sympathetic activation, catecholamine surges, impaired heart rate variability,^16^, ^17^ medullary respiratory dysfunction, neurogenic pulmonary edema, antiepileptic drug (AED) polytherapy, and ion channel dysfunction have all been implicated.^18^, ^19^, ^20^ Ion channelopathies such as catecholaminergic polymorphic ventricular tachycardia, Brugada syndrome, and long‐QT syndromes, can present with seizures, syncope, and arrhythmia.^21^, ^22^, ^23^, ^24^, ^25^, ^26^, ^27^, ^28^, ^29^, ^30^, ^31^, ^32^, ^33^, ^34^, ^35^, ^36^, ^37^, ^38^, ^39^, ^40^, ^41^ Several experts have proposed that "arrhythmogenic epilepsy" due to genetic or acquired ion channel disease (as a result of repeated seizures) could be predominant or contributing pathophysiological mechanisms in SUDEP.^18^, ^42^, ^43^

SUDEP is defined as a “non‐traumatic, non‐drowning, unexpected (witnessed or unwitnessed) death of an otherwise healthy PWE, which may or may not occur with evidence of an acute seizure (excluding status epilepticus).”^13^, ^14^ International guidelines have existed for many years recommending comprehensive investigation of PWE decedents to determine whether a SUDEP or non‐SUDEP mechanism (such as drowning, trauma, or suicide) may be responsible for death.^44^, ^45^

SUDEP guidelines have recommended specialist neuropathologist input to examine brain tissue, analogous to better established sudden cardiac death (SCD) autopsy guidelines that mandate performance in each case of drug and toxin screening, heart examination by cardiac pathologists, and blood storage for postmortem genetic testing.^46^, ^47^ Autopsy series in PWE have shown inconsistent systematic and comprehensive assessment,^48^, ^49^, ^50^, ^51^ prompting the publication of a recent position paper on the “Recommendations for the Investigation and Certification of Deaths in PWE” by the National Association of Medical Examiners, which included an expert panel of pathologists, medical examiners, epileptologists, and cardiologists.^52^

The aims of this study were to report characteristics of a large cohort of SUDEP cases from a tertiary care academic medical center, review consistency of evaluation of cases of SUDEP, and perform adjunct postmortem next generation whole exome sequencing (WES) to identify ion‐channel, arrhythmia, epilepsy‐related, and sudden death‐related variants.

## Methods

This study was approved by the institutional review board of The Mayo Clinic Rochester, MN; and informed consent was obtained.

### Mayo Clinic Sudden Death Registry

**Figure 1:**
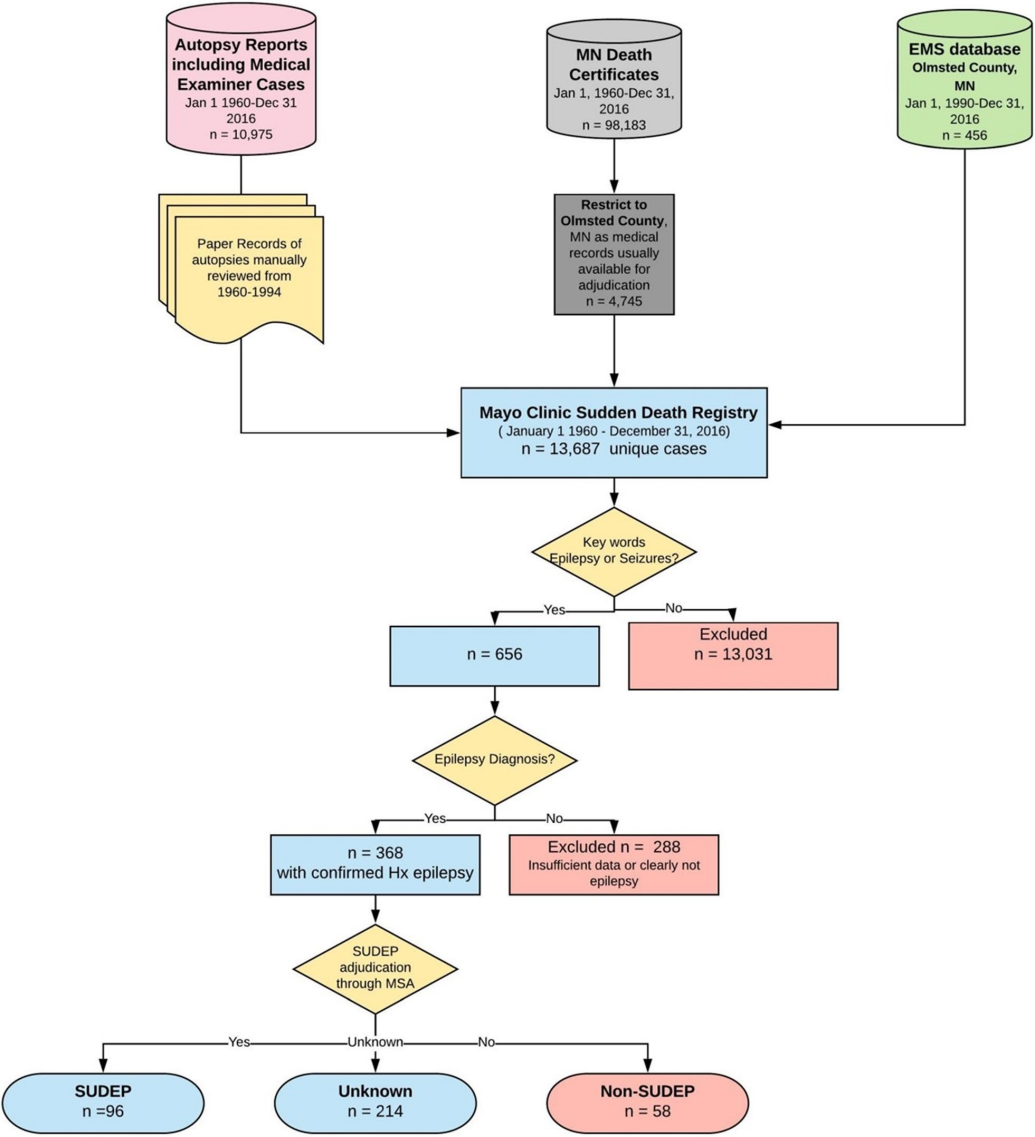
Case selection from the Mayo Clinic Sudden Death Registry. Multisource case ascertainment was used to build the registry including autopsy reports, medical examiner and coroner cases, death certificate searches using validated criteria, and EMS data. Cases were then selected based on a history of seizures or syncope and then adjudicated to determine if there was a diagnosis of epilepsy. Cases of secondary seizures, even if a sudden death event occurred, were excluded. The final cohort was then classified into SUDEP categories. EMS indicates emergency medical service; Hx, history; MN, Minnesota; MSA, multisource ascertainment; and SUDEP, sudden unexpected death in epilepsy.

For the purpose of this study, we built the MCSDR (Mayo Clinic Sudden Death Registry), which contains all cases (ages 0–90 years and both sexes) of sudden unexpected and unexplained deaths from 1960 to present that have been verified by multisource ascertainment of postmortem reports, county medical examiner’s office (or coroner) reports, validated death certificate data, and the emergency medical services (EMS) data (n=13 687; Figure 1). This includes SCD, sudden unexplained death syndromes, sudden infant death syndrome, and out‐of‐hospital cardiopulmonary arrest cases.

The definition of SCD is based on the World Health Organization criteria: sudden and unexpected death occurring within 60 minutes of the onset of symptoms (if witnessed) and within 24 hours if observed alive and symptom free (if nonwitnessed). Mayo Clinic Rochester performs autopsies on cases of sudden unexpected or unexplained death or deaths that occurred rapidly before a diagnosis could be accurately determined, or those occurring in the setting of recent medical intervention or surgery. All autopsies for the local area of Olmsted County, MN are conducted here, as are those performed for neighboring counties and the states of Minnesota, Wisconsin, Iowa, and New York.

Using the Minnesota Death Tapes, a statewide electronic record of death certificates, all potential sudden deaths cases have been identified with the use of validated search criteria (n=98 183 from January 1, 1960 to December 31, 2016).^53^, ^54^, ^55^ Given that medical records are available for >90% of Olmsted County residents at Mayo Clinic Rochester, we included only those with an Olmsted County address of residence (n=4745). Gold Cross is the sole EMS provider for Olmsted County and the surrounding area. Since January 1, 1990, a record of all arrests attended by the EMS has been maintained and these data were also incorporated into the MCSDR.

### Inclusion and Exclusion Criteria

We screened the MCSDR for all cases with keywords “seizure” or “epilepsy” between the dates of January 1, 1960 to December 31, 2016. Cases with the following were excluded: known malignancy, intracranial hemorrhage (extradural, subdural, subarachnoid, intraventricular or intraparenchymal), suicide, homicide, and status epilepticus.

Each case was then reviewed in detail, including paper and electronic records, death certificates, obituaries, autopsy reports, medical examiner reports, and EMS data, where available. A diagnosis of epilepsy was made based on International League Against Epilepsy criteria based on 1 or more of the following 3 criteria: (1) at least 2 unprovoked (or reflex) seizures occurring >24 hours apart; (2) 1 unprovoked (or reflex) seizure and a probability of further seizures; and (3) diagnosis of an epilepsy syndrome.^56^

### SUDEP Adjudication

Deaths were classified as non‐SUDEP when there was clearly an alternative cause of death such as trauma, drowning, drug overdose, suicide or homicide or as SUDEP cases as follows: (1) definite if the autopsy did not reveal a cause of death; (2) definite‐plus where an autopsy was performed and there was an alternative contributing factor; (3) probable in the absence of an autopsy but other supporting evidence; (4) possible in the absence of an autopsy and if there was a competing cause of death; and (5) unknown if there were no autopsy, no witness, and limited records.^13^, ^14^

### Case Note Abstraction

Medical, EMS, and autopsy reports were comprehensively and systematically reviewed and abstracted to an electronic case report form in REDCap (Vanderbilt University) hosted at Mayo Clinic.^57^ REDCap (Research Electronic Data Capture) is a secure, web‐based application designed to support data capture for research studies, providing (1) an intuitive interface for validated data entry, (2) audit trails for tracking data manipulation and export procedures, (3) automated export procedures for seamless data downloads to common statistical packages, and (4) procedures for importing data from external sources.

The following variables were abstracted: (1) demographics; (2) interviews with witnesses/family members; (3) emergency‐medical response team data; (4) medical records (including history of seizures, past medical history, medication use, smoking, alcohol and use of drugs of abuse, electrocardiography, electroencephalography, and imaging of the brain and heart); (5) coroner’s report including circumstances of death; (6) autopsy report including gross macroscopic and microscopic evaluation of the brain, heart, and lungs; and (7) toxicology screen. All entries were rechecked by a second abstractor and then 10% randomly checked to ensure consistency with an error rate of <2%.

### Postmortem Next Generation Whole Exome Sequencing

#### DNA Isolation

Genomic DNA was isolated from autopsy whole blood or frozen tissue using the Gentra Puregene Blood Kit (Qiagen, Germantown, MD) following the manufacturer’s protocol.

#### Whole Exome Next‐Generation DNA Sequencing

Genomic DNA samples were submitted to Mayo Clinic’s Advanced Genomics Technology Center for WES. The Bravo liquid handler and Aligent’s protocol was used to prepare paired‐end libraries, and DNA was fragmented using a Covaris E210 sonicator. Agencourt AMPure SPRI beads were used to purify the constructs. SureSelect forward and Agilent SureSelect ILM Pre‐Capture Indexing reverse primers were used to enrich the DNA fragment libraries, which were analyzed with Agilent Bioanalyzer DNA 1000 chip.

Exome capture was performed with the SureSelect XT Human All Exon V5 plus UTR Target Enrichment System (Agilent, Santa Clara, CA). Dynal Dynabeads MyOne Streptavidin T1 captured the DNA:RNA hybrids, and Agencourt Ampure XZP beads eluted DNA from the beads, which were amplified with Agilent Sure Select Post‐Capture Indexing forward and Index PCR reverse primers. Sequencing of the exome libraries was completed with Illumina HiSeq 2000 platform (San Diego, CA) and TruSeq SBS sequencing kit V3 reagents.

### Variant Filtering and Pathogenicity Assessment

Following WES, single nucleotide variants and insertion/deletions were filtered to identify variants that followed either a dominant or recessive inheritance pattern using Ingenuity Variant Software (Qiagen, Redwood City, CA). All variants within 71 epilepsy‐ (from Bagnall),^58^ 5 central hypoventilation‐ (from Bagnall),^58^ and 90 genetic heart disease (long‐QT syndrome, catecholaminergic polymorphic ventricular tachycardia, Brugada syndrome, hypertrophic cardiomyopathy, dilated cardiomyopathy, and arrhythmogenic right ventricular cardiomyopathy)—susceptibility genes (Table S1) were first filtered for a call quality score ≥20 and a read depth ≥10. Only nonsynonymous variants (NSV, ie, amino acid altering: missense, nonsense, splice‐error, frameshift insertion/deletions, or in‐frame insertion/deletions) were considered potentially pathogenic.

For the dominant model, only ultrarare variants (minor allele frequency [MAF] ≤0.00005) (1:20 000 alleles) in Genome Aggregation Database (gnomAD; http://gnomad.broadinstitute.org) were considered. Variants with a MAF >0.00005 in any ethnic group of gnomAD were excluded, unless observed only once in that ethnic group.

For the recessive inheritance model, only rare (MAF ≤0.01 in gnomAD) variants present as homozygotes were considered. Variants with a homozygous frequency >0.0001 in gnomAD were excluded from analysis. Given that parental DNA was unavailable to confirm that variants were in trans (on opposite alleles), compound heterozygotes (2 unique mutations in the same gene) were excluded from the recessive analysis. A comparison of yield of NSVs for both the dominant and recessive model was performed for all 166 sudden death‐susceptibility genes.

The American College of Medical Genetics and Genomics (ACMG) and Association for Molecular Pathology standards and guidelines for the interpretation of sequence variants were used to classify identified variants as pathogenic (P), likely pathogenic (LP), or variant of uncertain significance.^59^ Automatic variant classification was performed using InterVar, a freely available web‐based bioinformatics software tool for clinical interpretation of genetic variants by the ACMG/Association for Molecular Pathology 2015 guideline.^60^

### Statistical Analysis

All calculations were performed using SAS^®^ or JMP 13.0 (SAS Institute, Cary, NC). Continuous variables are reported via means with SD; categorical variables are reported as frequencies and percentages with associated CIs. All continuous variables were tested for normality and appropriate parametric tests used (eg, Shapiro‐Wilk or Kolmogorov‐Smirnov tests), as appropriate given distributional assumptions. For categorical variables, chi‐square tests or Fisher’s exact tests, and for nonnormally distributed data the appropriate nonparametric tests that met the nonparametric assumptions were used instead. For variant calling, Fisher’s exact tests were performed to determine statistical significance between 2 groups. All tests are 2 sided with an a priori comparison‐wise α level of <0.05. The first author had full access to all the data in the study and takes responsibility for its integrity and the data analysis.

## Results

A keyword search for “seizure” or “epilepsy” identified 656 (4.8%) of potential cases from the MCSDR of whom 288 (43.9%) either did not have epilepsy or had insufficient records to firmly establish diagnosis of epilepsy before sudden death. Many cases had secondary seizures, such as anoxic seizures following prolonged cardiac arrest times, seizures related to electrolyte abnormalities, seizures in the context of sepsis, meningoencephalitis, or toxins including alcohol‐related. The remaining 368 were confirmed to have epilepsy, either current or with a past diagnosis of epilepsy that is no longer present. Of these, 214 (66% male, mean age at death of 64.7±18.4, years) had only death certificate data and insufficient records to be classified as SUDEPs.

Where data were available, the majority had SCD events owing to acute myocardial infarction or arrhythmia in the presence of established heart failure. These were classified as unknown given the lack of information, it is unclear if they were possible SUDEPs. Of particular note, there was a single case of a 17‐year‐old girl with Aicardi syndrome (malformation syndrome with partial or complete absence of the corpus callosum associated with refractory seizures) who died at home, and a death certificate stated, “natural causes.” No specific details or an autopsy were available regarding the death, and hence our rationale was to classify this case as unknown, although it could possibly have been SUDEP.

**Figure 2:**
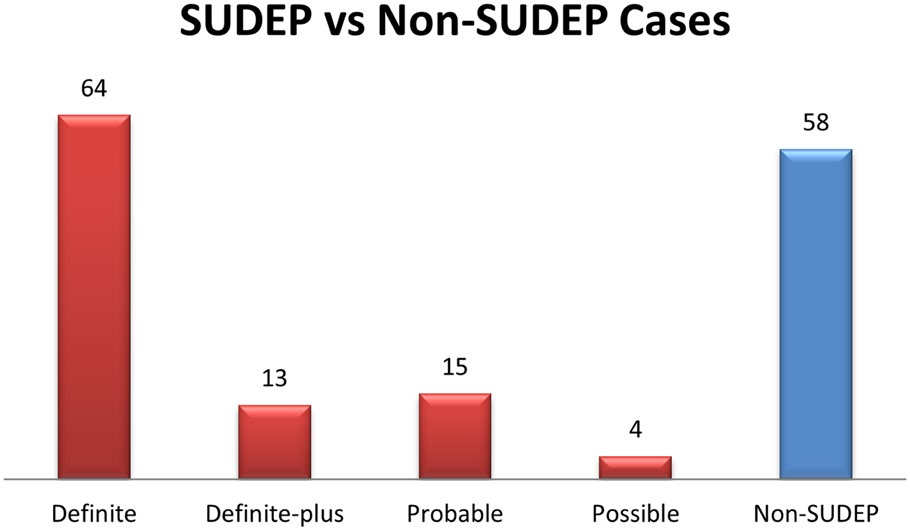
Classification of deaths in people with epilepsy, based on SUDEP Task Force and expert recommendations into definite, definite‐plus, probable, possible, and non‐SUDEP. SUDEP indicates sudden unexpected death in epilepsy.

Fifty‐eight cases (15.8%) were unequivocal non‐SUDEPs with causes including drowning, trauma, and poisoning. In addition, 96 cases (26.1%) were classified as definite, definite‐plus, probable, and possible SUDEP cases (Figure 2).

### SUDEP Versus Non‐SUDEP

The main clinical findings in the SUDEP group are summarized (Table 1). The mean age of SUDEP cases was 36.9 (±19.6) years and 57.5% were men (Table 2). The mean age of non‐SUDEP cases was significantly higher at 53.0 (±18.3) years (*P*<0.0001), with a similar male majority (69.0% men, *P*=0.1606). There was no difference in body mass index in SUDEP versus non‐SUDEP cases (*P*=0.2774).

**Table 1:** Clinical Characteristics of the SUDEP Cohort Combined and by Sex

Variable	Overall (n=96)	Male sex (n=56)	Female sex (n=40)	*P* value
Demographics				
From Midwest, n (%)	92 (95.8)	53 (94.6)	40 (97.6)	0.64
Resident of Olmsted County, MN	59 (61.5)	34 (56.7)	26 (43.3)	0.85
Race/Ethnicity, n (%)				
White	61 (64)	38 (67.8)	23 (57.5)	…
Native American or Alaska Native	1 (1)	0 (0)	1 (2.5)	…
Asian	1 (1)	0 (0)	1 (2.5)	…
Black	3 (3)	3 (5.4)	0 (0)	…
Hispanic	0 (0)	0 (0)	0 (0)	…
Native Hawaiian or Pacific Islander	0 (0)	0 (0)	0 (0)	…
Mixed race	3 (3)	2 (3.6)	1 (2.5)	…
Unknown or not disclosed	27 (28.1)	13 (23.2)	14 (35.0)	…
Age at epilepsy onset				
Mean±SD (range) y	21.2±17.2 (0–76)	21.1±17.5 (0–74)	21.3±17.0 (0–76)	0.48
Age at SUDEP				
Mean±SD (range) y	37.4±19.4 (2–84)	37.3±17.8 (3–80)	37.6±21.7 (2–84)	0.47
Body mass index				
Mean±SD (range), kg/m^2^	26.5±8.4	26.8±7.9	26.1±9.2	0.693
Duration of seizures				
Mean±SD (range) y	15.8±13.02 (0–57)	15.6±11.9 (0–45)	16.0±14.5 (0–57)	0.44
Recent onset (1–2 y), n (%)	22 (23.0)	12 (21.0)	10 (25.0)	…
Intermediate duration (3–10 y), n (%)	20 (21.0)	11 (20.0)	9 (23.0)	…
Chronic epilepsy (>10 y), n (%)	54 (56.0)	33 (59.0)	21 (53.0)	…
Etiology of epilepsy				
Epilepsy syndrome				
Idiopathic	18 (18.7)	8 (14.2)	10 (25.0)	…
Primary generalized epilepsy	8 (8.3)	3 (5.4)	5 (12.5)	…
Juvenile myoclonic epilepsy	1 (1.0)	1 (1.8)	0 (0.0)	…
Genetic syndromes				
Dravet	4 (4.2)	2 (3.6)	1 (2.5)	…
DiGeorge	0 (0.0)	0 (0.0)	0 (0.0)	…
Down	0 (0.0)	0 (0.0)	0 (0.0)	…
Structural/metabolic				
Posttraumatic epilepsy	5 (5.3)	5 (8.9)	0 (0.0)	…
Malformation of cortical development	7 (7.3)	5 (8.9)	2 (5.0)	…
Tumors/operated structural lesions	6 (6.3)	5 (8.9)	1 (2.5)	…
Temporal lobe epilepsy	3 (3.1)	0 (0.0)	3 (7.5)	…
Metabolic	1 (1.0)	1 (1.8)	0 (0.0)	…
Perinatal (ulegyria)	0 (0.0)	0 (0.0)	0 (0.0)	…
Postinfarct	2 (2.1)	2 (3.6)	0 (0.0)	…
Alcohol‐related	5 (5.2)	2 (3.6)	3 (7.5)	…
Immune‐related	2 (2.1)	0 (0.0)	2 (5.0)	…
Infectious	2 (2.1)	2 (3.6)	0 (0.0)	…
Mixed	9 (9.4)	5 (8.9)	4 (10.0)	…
Unclassified, n (%)	23 (23.9)	15 (26.8)	9 (22.5)	…
Seizure types				
Generalized, n (%)	51 (53)	30 (53.5)	21 (52.5)	…
Focal, n (%)	8 (8)	5 (8.9)	3 (7.5)	…
Focal and generalized, n (%)	11 (11)	6 (10.7)	5 (12.5)	…
Unspecified, n (%)	5 (5)	3 (5.3)	2 (5.0)	…
Duration of seizures	15.8±13.0	15.6±11.9	16.0±14.5	0.44
Mean±SD (range) y	(0–57)	(0–45)	(0–57)	
ECG performed, n (%)	15 (15.6)	…	…	…
Head magnetic resonance imaging/computed tomography data available, n (%)	13 (13.5)	…	…	…
Electroencephalography data available, n (%)	50 (52.1)	…	…	…
ECG abnormal, n (%)	3 (3.1)	…	…	…
Antiepileptic medications		…	…	…
No or not compliant, n (%)	7 (8.8)	3 (6.1)	4 (12.9)	
1 AED	40 (50.0)	23 (46.9)	17 (54.8)	
2–3 AED	27 (33.7)	17 (34.7)	10 (32.2)	
>3 AED	6 (7.5)	6 (12.2)	0 (0.0)	
Antiarrhythmic drugs	0 (0.0)	…	…	…
Smoking status (former or current)	12 (12.5)	…	…	…
Alcohol dependence	10 (10.4)	…	…	…

AED indicates antiepileptic drugs; and SUDEP, sudden unexpected death in epilepsy.

* As per definition, SUDEP does not include cases of status epilepticus.

**Table 2:** Characteristics of SUDEP Versus Non‐SUDEP Deaths in PWE

Variable	All (n=154)	SUDEP (n=96)	Non‐SUDEP (n=58)	95% CI	*P* value
Age at death, y	43.0±20.6 1–89	37.0±19.7 1–84	53.0±18.3 1–89	0.942–0.981	<0.0001
Male sex	96 (62.3)	56 (57.5)	40 (69.0)	0.316–1.524	0.1606
BMI	26.2±8.4	26.5±8.4	23.1±8.1	0.724–3.215	0.2774

Multivariable logistical regression with outcome SUDEP vs non‐SUDEP: Model 1: age and BMI 95% CI, 0.153–6.515 (*P*=0.9); Model 2: age and sex 95% CI, 0.295–2.861 (*P*=0.99); Model 3: age, sex, and BMI 95% CI, 0.053–18.71 (*P*=0.91). BMI indicates body mass index; PWE, people with epilepsy; and SUDEP, sudden unexpected death in epilepsy.

### Multisource Ascertainment

The records available for each class of SUDEP are summarized (Table S2). There were no emergency medical records for any of the decedents of SUDEP, either because an electronic database was only maintained from 1990, most of the cases died suddenly and unexpectedly, with electroencephalography‐confirmed epilepsy without postmortem or at the scene investigation, tended to be before the 1990s; deaths occurred outside of Olmsted County, MN, or as in the majority, decedents were already found dead.

### SUDEP Cases

The age range of decedents of SUDEP was 1 to 84 years with a mean (±SD) age of 37 (±19.7) years, and 56 (58.3%) were male. The majority were White (64%) and from the midwestern United States (95.8%). In 28.1%, ethnicity could not be accurately determined, particularly for cases with only paper records (before 1995), where this information was not consistently recorded. The mean age at epilepsy onset was 21.2 (±17.2) years and ranged from shortly after birth to age 76 years. The mean time from the diagnosis of epilepsy to SUDEP was 16.9±13.7 years. Six cases from the older age group tended to have structural etiologies such as postischemic stroke or postsurgery seizures, classed as epilepsy and requiring treatment.

### Etiology of Epilepsy

Based on the classifications of International League Against Epilepsy, the etiology of epilepsy was unknown in the majority. Cases of alcohol‐related seizures were excluded if clearly related to withdrawal and with occasional frequency. However, patients with likely underlying epilepsy who developed recurrent seizures in the absence of alcohol withdrawal or poisoning were included.

There were 4 cases of Dravet syndrome (DrS), 3 of whom were classified as definite SUDEPs, and 1 was classified as definite‐plus SUDEP. One was a 3‐year‐old girl of Native American descent with a de novo pathogenic variant in *SCN1A* (p.Asn359Ser) who had a history of febrile seizures and medically refractory afebrile generalized tonic‐clonic seizures and focal seizures. The patient had a witnessed cardiopulmonary arrest with a prolonged downtime, and although the patient was resuscitated, there was evidence of severe anoxic brain injury, and a decision was made to withdraw life support. The second case was a 2‐year‐old White boy with refractory myoclonic absence seizures and a de novo pathogenic variant in *SCN1A* (p.Arg101Gln). A 12‐lead ECG showed QTc at rest of 416 ms, and echocardiogram demonstrated a structurally normal heart. The patient was found dead in his crib in the prone position, and an autopsy revealed no abnormality. There were no significant findings with the other 2 cases of DrS with pathogenic variants in *SCN1A*, One 8‐year‐old White boy with de novo pathogenic variant in *SCN1A* (p.Arg786Val) and a 2‐year‐old Hispanic girl with a pathogenic variant in *SCN1A*.

### Circumstances Around Death

Only 15 (15.6%) SUDEP cases were witnessed, and the majorities of deaths occurred out of hospital, either at home or work (Table 3). Only 13 (13.5%) had a clearly documented seizure at the time of SUDEP or in the preceding 24 hours, although in the majority of cases, the presence or absence of a seizure proximate to death was not clearly documented.

**Table 3:** Circumstances of Death for 96 SUDEP Cases

	Overall	Male sex	Female sex
SUDEP category, n (%)	n=96	n=56	n=40
Definite SUDEP	63	38	25
Definite SUDEP‐plus	13	8	5
Probable SUDEP	15	8	7
Possible SUDEP	5	2	3
Witnessed death, n (%)			
Yes	15 (15.6)	9 (16.1)	6 (15.0)
No	56 (58.3)	36 (64.3)	20 (50.0)
Not documented	25 (26.1)	11 (19.6)	14 (35.)
Place of death			
Home or work	53 (55.2)	32 (57.1)	21 (52.5)
Nursing facility	6 (6.3)	5 (8.9)	1 (2.5)
In hospital	8 (8.3)	3 (5.4)	5 (12.5)
Other	3 (3.1)	2 (3.6)	1 (2.5)
Unknown	26 (27.1)	14 (25.0)	12 (30.0)
Evidence of preceding seizure activity before SUDEP			
Yes*	13 (13.5)	7 (12.5)	6 (15.0)
No	8 (8.3)	3 (5.4)	5 (12.5)
Unknown	75 (78.2)	46 (82.1)	29 (72.5)
Circumstances of death			
At rest	19 (19.8)	…	…
Suring emotional stress (bereavement)	1 (1.1)	…	…
During exercise	1 (1.1)	…	…
Nocturnal death	39 (40.6%)	…	…
Unknown	36 (37.4)	…	…
Position body was found in at time of death			
Prone	25 (26.0)	15 (26.8)	10 (25.0)
Supine	6 (6.3)	4 (7.1)	2 (5.0)
Indeterminate	65 (67.7)	37 (66.1)	28 (70.0)
Location of body			
Bed	19 (19.8)	…	…
Bedroom floor	4 (4.2)	…	…
Bathroom floor	4 (4.2)	…	…
Couch chair	2 (2.1)	…	…
Living room floor	6 (6.3)	…	…
No location documented	61 (63.5)	…	…

SUDEP indicates sudden unexpected death in epilepsy. Nocturnal death defined as occurring between 10:00 pm and 6:00 am.

* Includes a seizure the night or day before not exactly at the time of arrest.

In 60 cases having sufficient data to determine time of death, 39 (65%) occurred during the nighttime hours. Of the 21 daytime deaths, 19 occurred at rest, 1 during physical exercise, and 1 during emotional stress after receiving news of bereavement.

Of 35 cases with sufficient data regarding body location at time of death, 19 (54.3%) were found dead in bed, 4 (11.4%) were found on the bedroom floor, 4 (11.4%) were found on the bathroom floor, 2 (5.7%) were found slumped in a couch or chair, and 6 (17.1%) were found on the living room floor. In 31 cases with sufficient data for the body position found at death, 25 (80.6%) were found in the prone position, irrespective of location. Body location and position at the time of death were not recorded clearly in the remaining cases.

### Alcohol, Smoking, and Antiepileptic Drugs

A history of alcohol abuse could not be determined in 66 (68.8%) decedents. In the remaining 30, 10 (33%) had a history of alcohol dependence. Smoking status could not be determined in 70 (72.9%) cases, and of the remaining 26, 11 (42.3%) were either former or current smokers. None of the patients took cardiovascular drugs, and in particular no decedents were prescribed antiarrhythmic drugs. Fifty percent of decedents received 1 antiepileptic drug , 33.7% received polytherapy with 2 to 3 AEDs, and 7.5% received more than 3 AEDs.

### Postmortem Examination

Autopsies were performed in 83 cases, refused in 6, and unknown in 7. For the latter, it was not known whether an autopsy was performed as deaths occurred at locations outside of Olmsted County, MN, and may not have been referred to the local medical examiner or coroner. Three autopsies were performed at outside institutions in the vicinity of where death had occurred, and documentation indicated whether these were definite or definite‐plus SUDEP cases.

The majority of autopsies were performed within 12 to 24 hours of death, and 95.2% were complete (internal chest, abdomen, and neuropathological examinations). Two cases were outside referrals for specialist neuropathologist input (ie, brain tissue sent for further analysis, with general autopsy performed at a local center).^61^

Of the 83 cases with autopsies, 63 were classed as definite, 13 as definite‐plus, 6 as probable, and 1 as possible. The probable and possible cases either had limited autopsies, or the bodies had extensive decomposition, or there were other possible contributors.

### General Findings

Most autopsies (92%) were performed without embalming. Five (6.0%) had extensive autolysis, prohibiting detailed examination. All reports contained a general statement on external findings such as height, weight, and evidence of decomposition. Signs of trauma were reported in 7 (8.4%) cases, and not reported in 20 (24.1%). Regarding other external examination findings, only 8 (9.6%) had tongue or lip bite marks and 2 (2.4%) had petechiae possibly consistent with recent seizure activity. Only 1 (1.2%) had periorbital hematomas, possibly indicative of a fall and trauma to the face directly in the context of an arrest. Three (3.6%) had superficial burns with little other information provided (none of these cases had suspicion of deaths relating to fire). Seven (8.4%) had resuscitation marks, and of these, 3 were in witnessed SUDEP events, and the others were nonwitnessed deaths, but resuscitation was attempted.

### Neuropathology Findings

Thirty‐four (41.0%) autopsies had a neuropathologist examine the nervous system (Table S3). Detailed findings are in Data S1.

Regarding potential epileptogenic brain lesions, 6 (7.3%) had arteriovenous malformations (2 with telangiectasia). Other pathologies included cortical malformations in 10 (12.1%); focal cortical dysplasia in 4 (4.8%), and 1 (1.2%) case each of tuberous sclerosis, hemimegalencephaly, and grey matter heterotopia. The frequencies observed are similar to those reported in prior autopsy series.^48^, ^49^, ^50^, ^51^

Two cases had tumors, including a dysembryoplastic neuroepithelial tumor and an astrocytoma. Tumors in non‐SUDEP cases included 4 meningiomas and an astrocytoma. Old surgical scars were noted in 4 (4.8%) of SUDEP cases and included prior surgical treatments for psychoses and tumor removal with the subsequent development of a focal epilepsy syndrome.

Gross changes to the hippocampus were reported in 14 (16.9%) of cases. Microscopic hippocampal sclerosis was reported in 8 (9.6%) of cases, of which 6 were bilateral, and 1 case each for unilateral right sided and left sided. Two cases had both macroscopic and microscopic changes consistent with hippocampal malrotational abnormality. In 61 (73.5%) of cases, neither microscopic nor macroscopic changes were reported.

Regarding potential secondary consequences of seizures, old traumatic injuries were identified in 2 (2.4%), stroke in 3 (3.6%), mild cerebellar atrophy in 6 (7.3%), and severe cerebellar atrophy in 3 (3.6%) of cases. These are lower than reported in other series, but in cases extending back to the 1960s and1970s, findings may have been missed or not commented upon. Acute neuronal injury was present in 31 (37.4%) of cases; was located in the subiculum in 4 cases (extensive in 3) and in the cortex and basal ganglia in 11 cases; and its location was not described in the remainder.

### Cardiac Autopsy Findings

Thirteen (15.7%) had a cardiac pathologist examine the heart, or the full autopsy was performed by a cardiac pathologist who was on general duty. The mean heart weight was 358±138 g for all cases, and when restricted to adults was 392±110 g (Table S4). These are higher than published reference values (nondiseased hearts mean 331±56.7, range 233–383 g).^62^, ^63^ Five cases had left ventricular dilatation (mild in 4 and moderate in 1) and right ventricular dilatation (mild in 3 and moderate in 2). None of the cases had a diagnosis of heart failure syndrome antemortem or at autopsy. Hypertrophy of the right ventricle was reported in 2 (2.4%) and of the left ventricle in 14 (16.8%), which is higher than a previously reported frequency of 9.5%.^51^

The origin of the coronary arteries was reported in 43 (51.8%) of patients, including 1 case that had an anomalous left coronary artery arising from the noncoronary aortic sinus of Valsalva, which did not course within the aortic wall or between the aorta and pulmonary artery (which are established scenarios for sudden death).

In 1 case of DrS, a 23‐month‐old Hispanic girl suffered a witnessed asystolic sudden cardiac arrest and could not be resuscitated. The patient carried a familial pathogenic variant in *SCN1A* (exact details unavailable) and suffered from generalized and complex febrile seizures. Autopsy revealed an anomalous right coronary artery arising from the left aortic sinus of Valsalva, which coursed between the aorta and pulmonary artery, but with no evidence of infarction. The autopsy was at an outside institution and categorized as a SCD event. We instead categorized this case as SUDEP‐plus because it is plausible that the artery became compressed during a seizure or other stressful event. Witnesses of the arrest did not comment on the presence or absence of a seizure before the arrest.

Coronary artery atherosclerosis was present in 45 (54.2%) of decedents (Table S5), although absence of coronary artery atherosclerosis was not specifically commented on in 28 (33.7%) of cases. The majority were grade 1 (1–25% stenosed) or grade 2 (26–50% stenosed). However, 13 cases had grade 3 (51–75% stenosis) or grade 4 (75–100%) stenosis, which are considered clinically relevant to be classified as coronary artery disease (CAD), but only grade 4 severe CAD are acute coronary syndromes. Six decedents had single‐vessel CAD, 4 with 2‐vessel, 1 with 3‐vessel, and 2 with 4‐vessel CAD. No individuals had symptoms of angina, and none required any form of revascularization. Only 1 case had subendocardial myocardial infarction on microscopic evaluation.

One case of definite SUDEP had mitral valve prolapse without evidence of incompetence, which is associated with SCD.^64^ Three (3.6%) cases had bicuspid aortic valves. Two were detected at autopsy: 1 case was in a 16‐year‐old girl with fused right and left cusps and a shallow raphe, without evidence of stenosis or incompetence, who also had a left coronary artery arising from the noncoronary aortic sinus without an abnormal or high‐risk course. The second case also had a competent bicuspid valve with fusion of the right and left cusps. The third case had coarctation of the aorta and had undergone bioprosthetic replacement of the bicuspid valve as well as aortic repair. The subject had no myocardial injury, aortic damage, or endocarditis and died from a SUDEP event during sleep.

Two cases had slightly elevated blood alcohol levels. Most AED levels were either subtherapeutic or normal, and only 1 case had elevated blood levels of carbamazepine. There were no documented concerns or evidence for suicide or a possible contribution of alcohol, drugs, or prescription medications to the cause of death (Table S6).

### Consistency of Reports

We compared reporting to a standard set in an international guideline on investigation of deaths in PWE, which includes recommendations on information that should be included in SUDEP autopsy reports (Table S7). All reports had a brief history that was more detailed when a neuropathologist performed the entire autopsy or a brain‐only autopsy, and they tended to include details on seizure onset and type, as well as AED use. A full or partial autopsy was declined in 6 cases, but a report was generated based on available information as required by the medical examiner or coroner.

Signs of asphyxiation were commented on 71 (85.5%) of cases, and were seen in 4 (4.8%), all of which were classified as definite SUDEP as there was no evidence of another cause, suicide, or homicide. Only 1 pathologist commented on the presence of tuberous sclerosis, and there were no reported cases of Sturge‐Weber or neurofibromatosis.

### Sex‐Based Differences

Using sex as a biological variable, we compared differences between male and female cases (Table 1) and found no major differences specifically by region, age at epilepsy, age at SUDEP, duration of epilepsy, or mean number of AEDs.

### Antemortem Genetic Testing

Five of the decedent SUDEP cases had a diagnosis of Dravet syndrome with pathogenic variants identified in *SCN1A* gene. There were no other known monogenic disorders, and no cases with changes to copy number variants.

### Adjunct Postmortem Whole Exome Sequencing

Although blood was available on blood‐spot cards for cases from 1997 onwards, the Minnesota State Health Department had destroyed these following a state Supreme Court decision that these samples cannot be stored or used for research without informed consent.^65^ Furthermore, it has been routine to take whole blood at autopsy at Mayo Clinic Rochester since the 1980s, but if no further investigation is conducted in the absence of suspicion of unnatural deaths, then these samples are destroyed after 2 years. Although formalin‐fixed, paraffin‐embedded tissue is available, the yield of WES is typically poor and hence this was not used. Therefore, WES was performed on only 11 out of the 96 cases (6 White men, 1 White woman, 1 Black man, 2 Black women, and 1 Hispanic man).

### Yield of Ultrarare‐NSVs in Sudden Death‐Susceptibility Genes

Considering a dominant inheritance model, we identified 18 ultrarare (MAF <0.00005), NSVs within the 166 sudden death‐susceptibility genes in 6 (54.5%) of 11 SUDEP cases overall including 2 (66.7%) of 3 Black and 4 (57.1%) of 7 White decedents (Table 4). Furthermore, all 6‐variant positive SUDEP cases hosted multiple ultrarare NSVs amid these 166 genes. Considering a recessive inheritance model, homozygous or compound heterozygous variants were observed in 1 (9%) of 11 SUDEP cases. Following variant classification using the strict ACMG guidelines, none of the 19 variants observed in the 11 SUDEP cases achieved either a “pathogenic” or “likely pathogenic” designation. Therefore, all variants were instead classified as variants of uncertain significance.

**Table 4:** SUDEP Case Summary and Variant Adjudication for the 11 Cases Postmortem and 4 Cases Antemortem

Case	Sex	Age (y)	Race	Setting of SUDEP	Autopsy findings	Autopsy classification	Chromosome	Variant(s)‐gene	Associated disease(s)	SIFT	Polyphen	ACMG classification	Clinically actionable?
1	F	23	White	At rest in the bathroom	Cerebral swelling (mild) Grade I coronary artery disease	Definite SUDEP	10	**p.P412S‐CACNB2**	Brugada syndrome 4	Tolerated	Benign	VUS	No
							14	**p.A1611V‐MYH7**	HCM, DCM, LVNC	**Damaging**	Benign	VUS	No
							5	**p.A454T‐SDHA**	Leigh syndrome, Phaeochromocytoma, Nonsyndromic Paraganglioma	Tolerated	**Probably Damaging**	VUS	No
2	F	21	Black	Nocturnal	None Mild ethanol detected	Definite SUDEP	1	**p.T984A‐SZT2**	Epileptic encephalopathy	…	Benign	VUS	No
							1	**p.R1681H‐SZT2**	Epileptic encephalopathy	…	**Possibly Damaging**	VUS	No
							10	**p.E25del‐NEBL**	DCM, Liopatrophy,	…	…	VUS	No
							11	**p.R943Q‐MYBPC3**	HCM, DCM, LVNC	Damaging	…	VUS	No
3	M	48	White	Nocturnal	Unwitnessed sudden death Mild left ventricular hypertrophy	Definite SUDEP	1	**p.G1474E‐RYR2**	Catecholaminergic polymorphic ventricular tachycardia, arrhythmogenic right ventricular cardiomyopathy	…	**Possibly Damaging**	VUS	No
							3	**p.A756S‐PRICKLE2**	Myoclonic epilepsy	…	Benign	VUS	No
							7	**p.R900W‐CNTNAP2**	Cortical dysplasia focal epilepsy syndrome Autism	**Damaging**	**Possibly Damaging**	VUS	No
							18	**p.L451I‐MIB1**	LVNC	Tolerated	**Probably Damaging**	VUS	No
4	M	67	White	Unwitnessed	Mild focal coronary artery disease Macroscopically normal heart Microscopy patchy subendocardial fibrosis LV antero‐lateral and inferior wall	Definite SUDEP	8	**p.T163M‐CLN8**	Northern epilepsy	**Damaging**	**Probably damaging**	VUS	No
5	M	36	White	Nocturnal	Moderate cerebral swelling Chiari‐malformation type I Known generalized tonic‐clonic seizures (non‐compliant) Remote history ethanol misuse (toxicology screen negative) Found prone	Definite SUDEP	1	**p.V323M‐CNTN2**	Myoclonic epilepsy	**Damaging**	**Probably damaging**	VUS	No
							2	**p.V407L‐ST3GAL5**	Epilepsy	**Damaging**	Benign	VUS	No
							16	**p.R761Q‐ CACNA1H**	Familial hyperaldosteronism type 4, Childhood absence epilepsy, autism spectrum disorder	Tolerated	Benign	VUS	No
6	M	51	Black	Nocturnal	Witnessed seizure earlier in day Found dead prone in bed Negative toxicology screen Antiepileptic drugs subtherapeutic History coarctation aorta, Brachiocephalic‐arteriovenous, aorta repaired and functioning bioprosthetic valve Moderate left ventricular and right ventricular dilatation	Definite SUDEP	5	**p.P29S‐GDNF**	Hirschsprung disease 3, Pheochromocytoma, Congenital central hypoventilation	Tolerated	Benign	VUS	No
							6	**p.N68D‐LAMA4**	DCM	Tolerated	Benign	VUS	No
							16	**p.A1389T‐CACNA1H**	Familial hyperaldosteronism type 4, Childhood absence epilepsy, autism spectrum disorder	Tolerated	**Possibly Damaging**	VUS	No
							3	**p.R68H‐CLCN2** **(homozygous)**	Idiopathic generalized epilepsy childhood absence epilepsy	Tolerated	**Possibly damaging**	VUS	No
7	F	39	Black	Nonspecific	Tunneling of left anterior descending artery (1 mm deep)	Autopsy negative	…	…	…	…	…	…	…
8	M	39	Hispanic	Nocturnal	Intracranial arteriovenous malformations	Definite SUDEP	…	…	…	…	…	…	…
9	M	11	White	Nonspecific	History of sudden cardiac arrest during a witnessed seizure	Definite SUDEP	…	…	…	…	…	…	…
10	M	23	White	Nocturnal	Right frontal lobe cavernous malformations, cardiomegaly, alcohol and tetrahydrocannabinol abuse	Definite SUDEP	…	…	…	…	…	…	…
11	M	46	White	Diurnal	Cardiomegaly, sudden unwitnessed collapse in garage	Definite‐Plus SUDEP	…	…	…	…	…	…	…
12	M	8	White	…	…	…	2	**p.A786V** **SCN1A**	Dravet syndrome	…	…	LP	…
13	F	3	Native American	…	…	Definite SUDEP	2	**p.A359S** **SCN1A**	Dravet syndrome	…	…	LP	…
14	M	2	White	…	…	Definite SUDEP	2	**p.A101G** **SCN1A**	Dravet syndrome	…	…	LP	…
15	F	2	Hispanic	Diurnal	Anomalous coronary artery, Witnessed arrest without preceding seizure	Definite‐Plus SUDEP	2	**SCN1A**	Dravet syndrome	…	…	LP	…

The shaded cells distinguish an individual patient. The bold text refers to any variant which is potentially damaging.

ACGM indicates American College of Medical Genetics and Genomics; DCM, dilated cardiomyopathy; HCM, hypertrophic cardiomyopathy; LP, likely pathogenic; LV, left ventricle; LVNC, left ventricular noncompaction; SIFT, sorting intolerant from tolerant; SUDEP, sudden unexpected death in epilepsy; and VUS, variant of uncertain significance. The shaded cells distinguish an individual patient.

#### Case 1

A 23‐year‐old White woman hosted 3 variants (p.412S‐*CACNB2*, p.A1611V‐*MYH7*, and p.A454T‐*SDHA*). Although pathogenic variants in MYH7 are associated with hypertrophic cardiomyopathy, dilated cardiomyopathy, and left ventricular noncompaction and pathogenic variants in SDHA have been associated with Leigh syndrome (severe movement disorder), pheochromocytoma, and paragangliomas, the cardiovascular examination did not reveal any structural abnormalities, and there were no documented features consistent with Leigh syndrome, pheochromocytoma, or parangangliomas at autopsy.

#### Case 2

A 21‐year‐old Black woman hosted 4 ultrarare variants (p.T984A‐*SZT2*, p.R1681H‐*SZT2*, p.E25del‐*NEBL*, p.R943Q‐*MYBPC3*). Because parental DNA was unavailable, it is not known if the 2 *SZT2* variants are in cis (same allele) or trans (opposite alleles). Pathogenic variants in *SZT2* are associated with epileptic encephalopathy. Pathogenic variants in *NEBL* and *MYBPC3* have been associated with cardiomyopathies. However, the patient had a limited autopsy, so it is not known if there were structural abnormalities to the heart.

#### Case 3

A 48‐year‐old White man, who died during sleep, hosted 4 ultrarare variants (p.G1474E‐*RYR2*, p.A756S‐*PRICKLE2*, p.R900W‐*CNTAP2*, and p.L451I‐*MIB1*). Pathogenic variants in *RYR2* are known to cause catecholaminergic polymorphic ventricular tachycardia. Cardiac events related to catecholaminergic polymorphic ventricular tachycardia typically occur during exertion. Pathogenic variants in *PRICKLE2* are associated with myoclonic epilepsy and in *CNTNAP2* are associated with autism and cortical dysplasia focal epilepsy syndrome. It is unknown whether the decedent had myoclonic epilepsy, but he had no features of autism or cortical dysplasia focal epilepsy syndrome. Pathogenic variants in *M1B1* are associated with left ventricular noncompaction; however, the decedent showed no overt features of left ventricular noncompaction on autopsy.

#### Case 4

A 67‐year‐old White man hosted an ultrarare variant in *CLN8* (p.T163M‐*CLN8*). Pathogenic variants in *CLN8* have been associated with Northern epilepsy and neurodegenerative diseases typified by lipopigment accumulation. Although this was not specifically evaluated at autopsy, the decedent’s medical records did not suggest these features.

#### Case 5

A 36‐year‐old White man with definite SUDEP, hosted 3 ultrarare variants (p.V323M‐*CNTN2*, p.V407L‐*ST3GAL5*, and p.R761Q‐*CACNA1H*). Pathogenic variants in *CNTN2* and *ST3GAL5* are associated with myoclonic epilepsy and epilepsy, respectively. The third variant, *CACNA1H*, is a gene associated with autism and generalized absent seizures, which were not suggested by medical history.

#### Case 6

A 51‐year‐old Black man had coarctation of the aorta and a bicuspid aortic valve (both successfully repaired). He hosted 3 ultrarare heterozygous variants (p.P29S‐*GDNF*, p.N68D‐*LAMA4*, and p.A1389T‐*CACNA1H*) as well as a homozygous p.R68H‐*CLCN2* variant.

Pathogenic variants in *GDNF* are associated with central hypoventilation syndrome. The victim of SUDEP died at night and was found in the prone position, which is typical of central hypoventilation syndrome. He also had a history of alcohol dependence, and these 2 conditions may have contributed to SUDEP.

Pathogenic variants in *CACNA1H* are associated with autism and epilepsy, which the patient did not have. Pathogenic *LAMA4* variants are associated with dilated cardiomyopathy. The autopsy did reveal moderate left ventricular and right ventricular dilatation, which may reflect a dilated cardiomyopathy phenotype but equally could be because of prior coarctation repair and bicuspid aortic valve. The heart mass was 431 g (predicted 377 g for age and sex). It is unclear whether the aortic valve was stenotic, regurgitant, or both but did require surgery to repair. The valve and aorta had no other abnormalities at autopsy.

A homozygous p.R68H‐*CLCN2* variant was identified in SUDEP Case 6. The *CLCN2* gene encodes for chloride voltage‐gated channel 2. Heterozygous loss of function in *CLCN2* mutations with pathogenic variants have been implicated previously in idiopathic generalized epilepsies. The p.R68H‐*CLCN2* was reported previously in 1 of 35 African individuals from a combination therapy trial or longitudinal survey to evaluate immunological effects of malaria.^66^ Interestingly, p.R68H‐*CLCN2* was characterized functionally and shown to result in decrease steady state current and faster activation of chloride voltage‐gated channel 2 channels.

#### Cases 7–11

These exhibited multiple variants, but with our stringent cutoff, they did not meet criteria to be considered ultrarare (details are available on request).

## Discussion

This is one of the largest to date single‐center US series of SUDEP cases. It has 5 important findings:

1. Decedents of SUDEP tend to be younger than decedents of other causes in PWE, with the majority being unwitnessed and found in the prone position, consistent with other reports.^49^, ^50^, ^51^
2. Only 15% had an ECG performed, which may have missed coexistent channelopathies (long‐QT syndrome or Brugada syndrome).
3. Older subjects of SUDEP tended to have bystander coronary artery disease (without plaque events, chronic ischemia, or myocardial infarction), which could have been erroneously labeled as non‐SUDEP.
4. The most frequent antemortem genetic abnormality was pathogenic variants in the *SCN1A* gene associated with DrS.
5. Prior blood‐spot cards or postmortem blood samples had been discarded, thus eliminating a potential source to help elucidate genetic contributors and to illuminate mechanistic pathways contributing to SUDEP. Only 11 cases had sufficient samples to perform WES, which yielded no known pathogenic variants in arrhythmia, epilepsy, or respiratory‐related genes but did identify 18 ultrarare NSV variants in 6 cases.

Using the MSCDR, we identified 96 SUDEPs and 58 non‐SUDEPs, with the main difference being a younger age of death for the SUDEPs. The mean age of SUDEP decedents was 37.4 (±19.4) years, the young age at death underscoring the potential years of life lost in victims of SUDEP. Male sex was more frequent in both SUDEP and non‐SUDEP groups, with no statistically significant difference. ECGs were performed in only 15 cases, one of whom had a prolonged QTc interval with known DrS. The sheer lack of ECG data in the evaluation of cases with seizures and epilepsy likely results in the missing of important abnormalities because ECG remains the optimal way to phenotype patients for identifying coexisting channelopathies.

Autopsies reports were available in 83 cases and demonstrated no abnormal findings in just over 50%. Several cases were reported as SCD or CAD based on the presence of bystander CAD, when macroscopic and microscopic examination did not reveal acute or old infarction, or evidence of acute ischemia. None of the cases had angina when living, nor complained of chest pain just before death. Pulmonary edema was a frequent finding, either overt or microscopic, and is consistent with other reports, possibly representing neurogenic pulmonary edema in the setting of SUDEP. Table S8 summarizes prior SUDEP series and our findings. Autopsy reports since 2000 tended to be more systematic, including detailed cardiac and neuropathological evaluations.

One case of DrS had an anomalous coronary artery that coursed between the aorta and pulmonary artery, which could have become compressed during a seizure, leading to sudden death. This could be classified as a SCD or SUDEP, and without an autopsy would not have been identified, underscoring the importance of comprehensive autopsy examination. There was another case of an anomalous coronary artery with no abnormal course and therefore unlikely as a contributor to SUDEP. There were 3 (3.6%) cases of bicuspid aortic valve, which is higher than the population prevalence of 1%.^67^, ^68^

Antemortem genetic testing was only performed in cases suspected of DrS. However, several cases had other physical features (eg, hypertelorism) and severe intellectual disabilities that could point toward undiagnosed genetic disease. Most cases had been evaluated for known inborn errors of metabolism and were negative. However, with the advent of personalized medicine, these cases could be missed diagnostic odyssey cases, and identifying the genetic component could help shed light on SUDEP susceptibility in general. The frequency of known arrhythmia, seizures, and autonomic and SCD‐related genes in PWE are unknown. DrS is rare with an estimated incidence of 1:15 700 with most deaths occurring before 10 years of age nearly half because of SUDEP. Identifying DrS has implications for screening family members, providing prepregnancy counseling and possible prevention of SUDEP events using atropine as it can counter the parasympathetic hyperactivity observed in certain mice studies.^69^, ^70^, ^71^

In a large study from Australia of 61 SUDEP cases, the most frequent variants identified were in long‐QT syndrome genes (6 variants in *KCNQ1* and *KCNH2*), followed by 2 pathogenic variants in epilepsy‐related genes (*DEPDC5* and *PAFAH1B1*).^72^ The authors reported another 9 *de novo* variants described as pathogenic based on *in silico* prediction tools, but these may be classified as variants of uncertain significance based on ACMG criteria.

In our study we used a cutoff of a MAF <0.00005 to identify ultrarare variants (versus 0.1% in the aforementioned study). We did not detect known pathogenic variants or potential pathogenic variants in *DEPDC5* or *PAFAH1B1*. There are a number of possibilities for the observed low frequency of pathogenic variants, including low sample size for postmortem WES population differences from founder effects and differing criteria for variant calling. We did not detect any differences in copy number variants in our study, which have been reported in cases of autism with dup(15) and a high frequency for SUDEP.

SUDEP shares many similarities with sudden infant death syndrome and sudden unexplained death syndromes, including a high frequency of nocturnal deaths, being found in the prone position, negative autopsy findings, and a potential link to 5‐hydroxytryptamine.^73^ Most of our cases, where the position of the body was recorded, were prone.

## Conclusions

This is the one of the largest single‐center US series of SUDEP cases that used multisource ascertainment to determine the clinical profile, postmortem neuropathological, cardiovascular, and pulmonary findings. Several cases were classified as unknown deaths or SCDs and were adjudicated to be SUDEPs given the past history of epilepsy. Many cases had bystander cardiovascular findings that were insufficient to lead to death.

Antemortem genetic findings revealed *SCN1A* variants to be the most frequent contributor. Adjunct postmortem WES was performed in 11 cases and did not identify any known pathogenic variants relating to arrhythmia, respiratory, and epilepsy genes. Eighteen ultrarare variants were discovered and classified as variants of uncertain significance as per ACMG criteria.

The lack of available adequate blood or tissue for genetic analysis in this large‐scale series suggests that there remains a serious gap and urgent need for assembly of a large‐scale, multicenter, cooperative prospective sudden death registry in PWE to yield analyzable blood and tissue. These could better illuminate the multiple possible respiratory, cardiac, and epilepsy specific pathophysiologic mechanisms underlying SUDEP, so that the tragic consequences of SUDEP might be prevented in the future.

## Sources of Funding

This work was supported by the and National Center for Research Resources the National Center for Advancing Translational Sciences, National Institutes of Health, through Grant Number 1 UL1 TR002377 RR024150‐01. Chahal and Somers are supported by National Institutes of Health HL65176 and HL134885. Chahal is supported by the American Heart Association (Award number 17POST33400211). Ackerman and Tester are supported by the Mayo Clinic Windland Smith Rice Comprehensive Sudden Cardiac Death Program. The authors acknowledge support from the Mayo Foundation for Medical Education and Research, Department of Medicine Team Science Award. The contents of this article are solely the responsibility of the authors and do not necessarily represent the official view of the National Institutes of Health.

## Disclosures

No off‐label medication use. St. Louis reports that he receives research support from the Mayo Clinic Center for Translational Science Activities, supported by the National Center for Research Resources and the National Center for Advancing Translational Sciences, National Institutes of Health, through Grant Number 1 UL1 RR024150‐01.

Ackerman is a consultant for Audentes Therapeutics, Boston Scientific, Gilead Sciences, Invitae, Medtronic, MyoKardia, and St. Jude Medical. Ackerman and Mayo Clinic have an equity/royalty relationship with AliveCor, Blue Ox Health Corporation, and StemoniX. However, none of these entities were involved in this study in any way. Somers has served as a consultant for ResMed, Phillips, GlaxoSmithKline, Respicardia, Ronda Grey, Biosense Webster, Dane Garvin, Bayer, Itamar, and U‐Health. He works with Mayo Health Solutions and their industry partners on intellectual property related to sleep and to obesity. The remaining authors have no disclosures to report.

## Supporting information

Data S1
Tables S1–S8
