# Supplementary material for: Confirmation of Cause of Death Via Comprehensive Autopsy and Whole Exome Molecular Sequencing in People With Epilepsy and Sudden Unexpected Death
Source: J Am Heart Assoc. 2021 Nov 24;10(23):e021170. doi: 10.1161/JAHA.121.021170 (PMC9075361; doi:10.1161/JAHA.121.021170)
Supplement: Supplementary file 1 — Data S1 Tables S1–S8 [file JAH3-10-e021170-s001.pdf]

SUPPLEMENTAL MATERIAL

Data S1.

Supplemental Autopsy Findings

Neuropathology Findings: 34 (41.0%) autopsies had a neuropathologist examine the nervous system (**Table S3**). The mean mass of brains was 1329 (range 358-1720) g for all ages. The mean for decedents aged ≥ 18 years was 1384 (±147.6; range 1010-1720) g, which is similar to published normal reference ranges (mean 1407 g; range 1070-1767 g).[61] Brain mass was higher for cases with documented swelling vs. those without, but the difference was not significant (p=0.14). Twenty-four (28.9%) had carotid artery stenosis: grade I in 19, grade II in 2, grade III in 2, and grade IV in 1 case. Eight cases (9.6%) had hippocampal abnormalities, and absence of hippocampal abnormalities was documented in only 6 (7.3%), with no comment on the remaining 83.1%. Brain swelling was present in 12 (14.5%) cases, contusions in 4 (4.8%), and focal infarctions in 3 (3.6%).

Microscopic evaluation of the myocardium was reported in 43 (51.8%) of cases. Focal fibrosis was observed in 14 (32.6%) of cases, and most were described as non-specific. In 2 cases, fibrosis was considered to represent old myocarditis, and in another 2 cases there was patchy subendocardial fibrosis in a distribution inconsistent with infarction. Two cases had prominent but non-specific fatty infiltration of both the LV and RV.

Pulmonary Autopsy Findings: Lung mass was reported in 59 cases, with 527 ± 199 g (range 115-950 g) for the right lung and 468 ± 177 g (95-880 g) for the left lung. When restricted to adults, this was 568 ± 167 g (range 225-950 g) for the right lung and 508 ± 146 g (range 220-880 g) for the left lung, which are higher than published reference values.[61, 65] Pulmonary edema

was present in 27 (44.3%) of cases, and myocardial disease, renal disease, and heart failure were absent in all. Aspiration of gastric contents was noted in 5 (8.2%) of cases, 3 of which were classified as probable or possible cases of SUDEP. Two definite SUDEP cases had very aspiration, which often results from resuscitation or moving the body after death.

Postmortem Toxicological Studies: Toxicological screening was specifically documented in 62 (74.7%) cases, and positive for 15. For cases in the late 1970s and early 1980s, toxicological screening became increasingly utilized, and by the 1990s was reported in all cases. The breadth of toxins became extended coinciding with the greater availability of mass spectrometry, and includes drugs of abuse, prescription medications (opioids and benzodiazepines), and salicylate and acetaminophen (for suspected suicide overdose cases). The AED levels focused on prescribed drugs, with levels usually within normal range or subtherapeutic concentrations.

Height was recorded in 64 (77.1%) and weight in 65 (78.3%). Nourishment status was documented in 66 (79.5%) of cases, and reported as malnourished in 2 cases, both having a history of alcohol dependence. External injuries were reported on 63 (75.9%) of cases. Organs were examined by the pathologist prior to evisceration in 13 (15.6%) of cases.

Table S1. Hugo Gene Symbols of Candidate Genes Interrogated Following Postmortem Whole Exome Sequencing.

ALDH7A1	*SCN9A*	*ACTN2*
ARHGEF9	*SIAT9*	*ANKRD1*
ARX	*SLC13A5*	*BAG3*
ASAH1	*SLC25A22*	*CALR3*
CACNA1H	*SLC2A1*	*CRYAB*
CACNB4	*SNIP1*	*CSRP3*
CASR	*SPTAN1*	*CTNNA3*
CARS2	*SRPX2*	*DES*
CDKL5	*ST3GAL3*	*DSC2*
CHD2	*STRADA*	*DSG2*
CHRNA2	*STXBP1*	*DSP*
CHRNA4	*SYN1*	*DTNA*
CHRNB2	*SYNGAP1*	*EYA4*
CLCN2	*SZT2*	*FHL2*
CLN8	*TBC1D24*	*FKTN*
CNTN2	*TNK2*	*GATAD1*
CNTNAP2	*ASCL1*	*JPH2*
CPA6	*BDNF*	*JUP*

CSTB	*EDN3*	*LAMA4*
DEPDC5	*GDNF*	*LDB3*
DOCK7	*RET*	*LMNA*
DNM1	*KCNQ1*	*MIB1*
EFHC1	*KCNH2*	*MTO1*
EPM2A	*SCN5A*	*MYBPC3*
GABRA1	*RYR2*	*MYH6*
GABRB3	*AKAP9*	*MYH7*
GABRD	*ANK2*	*MYL2*
GABRG2	*CACNA1C*	*MYL3*
GNAO1	*CACNA2D1*	*MYLK2*
GOSR2	*CACNB2*	*MYOM1*
GRIN2A	*CALM1*	*MYOZ2*
HCN1	*CALM2*	*MYPN*
IER3IP1	*CALM3*	*NEBL*
KCNB1	*CASQ2*	*NEXN*
KCNA1	*CAV3*	*PDLIM3*
KCNMA1	*DPP6*	*PKP2*
KCNQ2	*GJA1*	*PLN*
KCNT1	*GPD1L*	*PRDM16*
KCTD7	*HCN4*	*PRKAG2*

LGI1	*KCND3*	*PSEN1*
ME2	*KCNE1*	*PSEN2*
MEF2C	*KCNE2*	*RBM20*
NECAP1	*KCNE3*	*SDHA*
NHLRC1	*KCNJ2*	*SGCD*
PAFAH1B1	*KCNJ5*	*TAZ*
PCDH19	*KCNJ8*	*TCAP*
PLCB1	*RANGRF*	*TGFB3*
PNKP	*SCN1B*	*TMEM43*
PRICKLE1	*SCN3B*	*TMPO*
PRICKLE2	*SCN4B*	*TNNC1*
PRRT2	*SNTA1*	*TNNI3*
SCARB2	*TRDN*	*TNNT2*
SCN1A	*AARS2*	*TPM1*
SCN2A	*ABCC9*	*TTN*
SCN8A	*ACTC1*	*TXNRD2*
		VCL

Table S2. Multi-source ascertainment of SUDEP cases based on classification by Nashef *et al*.

Classification	n	Autopsy or Medical Examiner Report	Death Certificate	EMR	Medical Records
**Definite SUDEP**	63	63	63	0	57
**Definite SUDEP-plus**	13	13	13	0	13
**Probable SUDEP**	15	6	15	0	14
**Possible SUDEP**	5	1	5	0	5
**Total**	96	83	96	0	89

SUDEP = Sudden Unexpected Death in Epilepsy

Table S3. Neuropathological Findings at Autopsy Examination for the 83 cases where autopsy reports are available.

Finding	Yes	No	Unknown or not documented
**Heart examined by cardiac pathologist**	13 (15.7)	45 (54.2)	25 (30.1)
**Brain examined by neuro-pathologist**	34 (41.0)	28 (34.0)	21 (25.0)
**Body embalmed**	7 (8.4)	54 (65.1)	22 (26.5)
**External**			
Signs of trauma	7 (8.4)	56 (67.5)	20 (24.1)
Tongue/lip biting	8 (9.6)	47 (56.6)	28 (33.7)
Petechiae	2 (2.4)	40 (48.3)	41 (49.3)
Periorbital hematoma	1 (1.2)	47 (56.6)	35 (42.2)
Burns	3 (3.6)	67 (80.7)	13 (15.7)
Resuscitation marks	7 (8.4)	47 (56.6)	29 (35.0)
**Macroscopic Brain Examination**			
Hippocampal abnormality	2 (2.4)	6 (7.3)	75 (90.3)
Mild brain swelling	12 (14.5)	44 (53.0)	27 (32.5)
Contusions	4 (4.8)	49 (59.0)	30 (36.2)
Evidence of infarction	3 (3.6)	48 (57.8)	32 (38.6)
Brainstem	40 (48.2)	5 (6.0)	38 (45.8)

**Categories of lesions with potential for epileptogenicity**			
AV malformations	6 (7.2)	47 (56.7)	30 (36.1)
Malformations of cortical development	7 (11.7)	--	--
FCD type IIB	4 (4.8)	42 (50.6)	37 (44.6)
Tuberous sclerosis	1 (1.2)	46 (55.4)	36 (43.4)
Hemimegalencephaly	1 (1.2)	45 (54.2)	37 (44.6)
Grey matter heterotopia	1 (1.2)	45 (54.2)	37 (44.6)
Polymicrogyria	0 (0.0)	45 (54.2)	38 (45.8)
Ulegyria/perinatal cortical infarct (+ associated FCDIIId)	0 (0.0)	45 (54.2)	38 (45.8)
Other FCD types (FCD I and mild MCD)	0 (0.0)	45 (54.2)	38 (45.8)
Aicardi syndrome	0 (0.0)	45 (54.2)	38 (45.8)
**Tumor lesions**			
DNT, oligodendroglioma, PA, meningioma, astrocytoma II, ganglioglioma	2 (2.4)	45 (54.2)	36 (43.4)
Old surgical scars	4 (4.8)	36 (43.4)	43 (51.8)
**Hippocampal sclerosis (confirmed on histology)**	8 (9.6)	14 (16.9)	61 (73.5)
Unilateral right	1 (1.2)	--	--
Unilateral left	1 (1.2)	--	--
Bilateral	6 (7.3)	--	--

HIPMAL (macroscopic and microscopic)	2 (2.4)	--	--
**Secondary neuropathology (sequelae of seizures)**		--	--
Old traumatic brain injury/contusions	2 (2.4)	43 (51.8)	38 (45.8)
Prior stroke	3 (3.6)	42 (50.6)	38 (45.8)
Mild cerebellar atrophy (microscopic)	6 (7.3)	39 (46.9)	38 (45.8)
Severe cerebellar atrophy (macroscopic)	3 (3.6)	42 (50.6)	38 (45.8)
**Evidence of Acute Neuronal Injury (AEN)**	31 (37.4)		
CA1/subiculum	4 (4.8)	41 (49.4)	38 (45.8)
Other location (cortex, basal ganglia)	11 (13.2)	34 (50.0)	38 (45.8)
Extensive changes	3 (3.6)	42 (50.6)	38 (45.8)

*Excludes aspiration

†Localized pathological lesions include causal as well as sequelae of seizures, however, excludes diffuse changes such as acute eosinophilic neurones

MCD=Malformations of Cortical Development

VM=Vascular malformations (and includes arterio-venous malformations and telangiectasias)

FCD=Focal Cortical Dysplasia

DNT=Dysembryoplastic Neuroepithelial Tumor

PA=Pilocytic Astrocytoma

HIPMAL=HIPpocampal MALrotational abnormality

AEN=Acute Eiosinophilic Neurones

Table S4. Cardiovascular and Pulmonary Findings at Autopsy.

**Cardiovascular**		
**Heart weight**	358 ± 138 g<br>(50-800 g)	Notes
**Pericardial abnormalities**	70 (84.3)	
**Great vessel abnormalities**	0 (0.0)	
**Coronary artery dominance**		
Right	51 (78.5%)	
Co-dominant	2 (3.1)	
Left	3 (4.6)	
Unknown	9 (13.8)	
Normal course	44	
CAD Grade III-IV		
**Myocardium**		
Evidence of focal fibrosis	14 (32.6)	Microscopy reported in 41 cases
Evidence of infarction	1 (2.3)	
Fatty infiltration	2 (4.7)	
LV dilatation	5 (6.0)	

LVH	14 (16.9)
RV dilatation	5 (6.0)
RVH	2 (2.4)
Pericardium abnormal	0 (0.0)
Significant pericardial fluid	0 (0.0)
**Valves**	
MV prolapse	1 (1.2)
Mitral regurgitation	0 (0.0)
Bicuspid aortic valve	3 (3.6)
Aortic stenosis	0
Aortic regurgitation	0
**Conduction System Disease**	
SA node fibrosis	0
AV node fibrosis	1
Pulmonary Findings	
Right lung mass	527 ± 199 g<br>(115-950)

Left lung mass	468 ± 177 g<br>(95-880)
COPD changes	
Pulmonary edema/congestion*	27 (44.3)
Aspiration of gastric contents?	5 (8.2)
Tumors	0 (0.0)
Pulmonary embolism	0 (0.0)

CAD = Coronary Artery Disease

SA node = Sino-Atrial node

RVH = Right Ventricular Hypertrophy

LVH = Left Ventricular Hypertrophy

Table S5. Atherosclerotic Coronary Artery Disease.

	Grade 0	Grade 1 (1-25%)	Grade 2 (26-50%)	Grade 3 (51-75%)	Grade 4 (>75%)	Unknown
**RCA**	15	27	9	3	1	28
**LCA**	18	25	6	1	1	32
**LAD**	11	24	10	7	4	27
**LCX**	11	30	3	4	4	31
**Sum**	55	106	28	15	10	118
**Significant CAD > 75% stenosis)**						
Single-vessel	6 (10.2)					
Two-vessel	4 (6.8)					
Three-vessel	1 (1.7)					
Four-vessel	2 (3.4)					

RCA = Right Coronary Artery

LCA = Left Coronary Artery

LAD = Left Anterior Descending Artery

LCX = Left Circumflex Artery

Table S6. Toxicological Studies and Anti-Epilepsy Drug Levels at Autopsy.

Toxin	Not detected	Within normal, safe or therapeutic range	AED sub-therapeutic	Higher than usual	Unable to determine or not tested
**Alcohol (Ethanol)**	32	3	-	2	46
**Salicylate**	24	1	-	0	58
**Acetaminophen**	25	1	-	0	57
**Phenobarbital**	27	2	2	0	52
**Phenytoin**	8	4	3	0	53
**Valproate**	23	3	0	0	57
**Lamotrigine**	24	1	0	0	58
**Carbamazepine**	25	1	1	1	55
**Hydroxycarbazepine**	24	0	0	0	59
**Primidone**	24	0	0	0	59
**Cocaine**	30	-	-	-	53
**Cannabis**	29	-	-	-	54
**Narcotic analgesics***	28	2	-	1	52
**Stimulant analgesics****	30	-	-	-	53
**Benzodiazepines*****	27	2	-	-	54

**Anti-depressants**	18	1	-	1	63
**Environmental toxins**	24	-	-	-	59

*Narcotic analgesics include codeine, methadone, pethidine, morphine, hydrocodone, oxycodone, fentanyl

**Stimulants Include Amphetamine, methamphetamine, MDMA (ecstasy), pseudoephedrine, fenfluramine, phentermine

***Includes diazepam , alprazolam, zolpidem , zopiclone, zaleplon

Table S7. Consistency of Autopsy Reports.

	Recorded in n (%)	Mean ± SD (range)
History added to report	83 (100)	
Autopsy permission declined	6	
**External examination**		
Height mean ± SD cm	64 (77.1)	164.8 ±20.7
Weight mean ± SD kg	65 (78.3)	76.6 ± 35.2
Nourishment status	66 (79.5)	
External injuries commented	63 (75.9)	
Body inspected by pathologist prior to evisceration?	13 (23.2)	
Signs of asphyxiation?	71 (85.5)	
Conditions associated with epilepsy		
Neuro-fibromatosis	0 (0.0)	
Sturge-Weber	0 (0.0)	
Tuberous sclerosis	1 (1.2)	
Full autopsy performed	79 (95.2)	
**Blood collected for genetic studies**		
Whole blood tubes	0 available	
Blood-spot cards	11	

**Brain (as per international guidelines)**[41,41]		
**Mass of brain**	69 (83.1)	1329.4 (±256.2) g
Swelling	56 (67.5)	
Contusions	53 (63.9)	
Developmental abnormalities	53 (63.9)	
Mesial temporal (Ammon's horn) sclerosis	51 (61.4)	
Cerebellar atrophy	52 (62.7)	
**Coronal slices**	Unable to determine	
Microscopy commented	79 (95.2)	
Brain sites for histology		
a) Cingulate gyrus	0 (0)	
b) Hippocampus and parahippocampal gyrus Right and Left	7 (8.4)	
c) Middle frontal gyrus	6 (7.2)	
d) Superior and middle temporal gyri	6 (7.2)	
e) Insula	4 (4.8)	
f) Caudate nucleus	4 (4.8)	
g) Putamen and globus pallidus	5 (6.0)	
h) Pons	6 (7.2)	
i) Cerebellar vermis	12 (14.5)	

j) Cerebellar hemisphere including dentate nucleus	10 (12.0)
k) Brainstem	40 (48.2)
**Skin collected for possible molecular studies**	0 (0)
**Hair (for confirming AED compliance)**	0 (0)
**Heart (as per sudden cardiac death guidelines)[44]**	
Weight	71 (85.5)
Pericardium	70 (84.3)
Great vessels commented	58 (69.9)
**Coronary arteries**	
Origin	43 (51.8)
Dominance reported	65 (78.3)
Course of epicardial coronary arteries	44 (53.0)
**CAD**	56 (67.5)
**Myocardium and chambers**	
Microscopy reported	43 (51.8)
LV dilatation reported	56 (67.5)
LV hypertrophy reported	59 (71.1)
RV hypertrophy reported	59 (71.1)
RV dilatation reported	55 (66.3)

Fatty infiltration reported	2 (2.4)
Pericardium and effusion reported	59 (71.1)
**Valves**	
MV prolapse reported	67 (80.7)
Bicuspid aortic valve reported	62 (75.0)
Tricuspid atresia reported	0 (0.0)
Aortic stenosis reported	62 (75.0)
**Conduction system**	
Examined	2 (2.4)
SA node reported	2 (2.4)
AV node reported	2 (2.4)
**Lungs**	
Right lung mass	59 (71.1)
Left lung mass	59 (71.1)
Pulmonary edema/congestion	61
Aspiration of gastric contents	61 (73.5)
Tumors	0 (0.0)
Pulmonary embolism	61 (73.5)
**Toxicological analyses**	62 specifically documented

AED=anti-epileptic drugs

Table S8. Characteristics of Individual SUDEP Cases.

Case number	Sex	Age at Death	SUDEP classification as per Nashef 2012	Anti epileptic drugs	ECG performed	Heart rate (bpm)	Rhythm	QTc	Comments ECG	Position at time of death prone supine unknown	Time of death day night unknown	Comments	Gene
1	F	23	Definite	Valproic acid						Unknown	Night	Cerebral swelling, diffuse, mild; history of chronic seizure disorder	
2	F	21	Definite							Unknown	Night	Hx of seizure disorder and recent ethanol ingestion	
3	M	48	Definite	Phenytoin, carbamezapine						Unknown	Night	Hx seizure disorder and chronic alcoholism	
4	M	67	Definite							Unknown	Night	Hx seizure disorder and coronary atherosclerosis	
5	M	36	Definite	Valproic Acid, phenytoin	yes	76	Sinus rhythm	441	Normal ECG	Unknown	Day	Hx GTCS	
6	M	51	Definite	Topiramate, levetiracetam						Prone	Day	Hx coarctation of aorta, aortic stenosis, bicuspid aortic valve, ascending aortic aneurysm	
7	M	48	Definite	Valproic acid, phenobarbital						Prone	Night	Juvenile myoclonic epilepsy	
8	M	38	Definite	Phenobarbital						Unknown	Night	Vascular malformations	
9	F	11	Definite	Lamotrigine, levetiracetam, diazepam						Unknown	Unknown	Hx seizure disorder, ADHD	
10	M	23	Definite	Diazepam, levetiracetam						Unknown	Day	Hx alcohol and tetrahydrocannabinol abuse	
11	M	46	Definitie-PLUS	Gabapentin, valproic acid, diazepam						Unknown	Day	Cardiomegaly, follicular adenoma of the thyroid gland	
12	F	35	Definite	Phenytoin, diazepam, valproic acid, phenobarbital						Unknown	Day	Cerebral plasy	
13	F	38	Definite	Phenobarbital, diazepam						Prone	Night	h/o focal and generalized seizures	
14	M	26	Definite	Carbamezapine, phenobarbitol						Prone	Day		
15	M	49	Definite	Phenobarbital, diazepam, phenytoin						Unknown	Unknown	Temporal lobe seizures for 10+ years; hippocampal sclerisis	
16	F	41	Definite							Unknown	Day	h/o Sarcoidosis	
17	F	31	Definite	Phenytoin						Prone	Unknown		
18	F	40	Definite	Phenobarbital, phenytoin						Unknown	Day	Hx GTCS 24yrs	
19	M	17	Probable	Valproic acid, phenobarbital, carbamezapine						Unknown	Night	Cerebral palsy, mental retardation	
20	M	7	Probable	Carbamezapine	yes					Unknown	Day	Severe hydrocephalus; convulsive disorder,focal seizures	
21	M	72	Possible	Phenytoin, phenobarbital	yes		Sinus rhythm		Anterior Myocardial infarc	Unknown	Unknown	Central arteriosclerosis and convulsive disorder, Hx of focal and generalized seizures	
22	F	20	Probable	Phenytoin						Unknown	Unknown	Profound MR, motor deficits, spastic hemiplegia	
23	M	33	Probable	Phenobarbital						Unknown	Unknown		
24	F	32	Unknown	Phenytoin, phenobarbital						Unknown	Unknown		
25	M	27	Probable	Phenobarbital, phenytoin	yes	123	Sinus tachycardia		Poor R wave progression	Unknown	Unknown	Hx focal and generalized seizures	
26	M	23	Probable	Phenytoin, carbamezapine, phenobarbital	yes	107	Sinus tachycar	421	Left ventricular hypertroph	Unknown	Night	seizure/seizure disorder	
27	M	31	Definite	Phenobarbital	yes	56	Sinus bradycar	413	Normal ECG	Prone	Night	Genrealized seizure disorder, posturally dependent obstructive sleep apnea, congenital heart disease (bicupsid aortic valve, ventricular se	
28	M	30	Definite	Phenytoin						Prone	Unknown	History of several surgical procedures	
29	F	79	Definite	Phenytoin						Prone	Day	Temporal lobe epilepsy; chronic bronchitis, bilateral pulmonary edema; microscopic bone marrow and fat emboli (focal)	
30	F	26	Definite	Phenobarbital, phenytoin, lamotrigine, valproic acid						Prone	Night	Achondroplastic dwarfism, obesity	
31	F	7	Definite	Carbamezapine, valproic acid, phenobarbitol						Unknown	Night	Hydrocephalus secondary to aqueductal stenosis (VP shunt in place), developmental abnormalities (partial agenesis of corpus callosum,	
32	F	15	Definite	Phenobarbital, clonazepam						Prone	Night	Generalized seizure disorder; previous infantile meningitis	
33	F	51	Definite	Carbamezapine, phenytoin						Unknown	Day		
34	M	53	Definite	Phenytoin, phenobarbital						Prone	Day	nodular heterotopia, focal, right perventricular areaa at level of rostrum of corpus callpsum, tathke's cleft remnants	
35	M	36	Definite	Phenytoin, gabapentin						Prone	Day	Marked bilateral hippocampal sclerosis without ventricular dilation; history of epileptic seizures since age 11 months	
36	F	32	Definite	Valproic acid						Unknown	Night	History of post-transfusion hepatitis C; previous motor vehicle accident with severe closed head injury (operated); congestion and edema	
37	M	44	Unknown	Lamotrigine						Unknown	Unknown		
38	F	3	Probable	Phenobarbital						Unknown	Day		
39	F	18	Probable							Unknown	Unknown		
40	F	34	Definite	Diazepam, Phenytoin, Clonazepam, Valproic Acid						Prone	Day	Intractable epilepsy, focal partial seizures	
41	M	33	Definite	Phenytoin, phenobarbital						Unknown	Night	Mental retardation, Mood disorder	
42	M	35	Unknown	Alprazolam, lamotrigine						Unknown	Unknown		
43	F	21	Definite	Phenytoin, phenobarbital						Unknown	Day		
44	F	74	Possible	Phenytoin, Diazepam						Unknown	Day	Idiopathic seizures, Immunosupression, Hypertension	
45	F	52	Definite	Phenytoin, phenobarbital						Prone	Night	Multiple sclerosis, Ischemic heart disease	
46	M	5	Definite							Prone	Day		
47	M	38	Definite							Prone	Day	Grand mal seizures, mental retardation	
48	M	31	Definite	Phenytoin						Unknown	Unknown	Focal subarachnoid hemorrhages, focal ischemia of ascending colon	
49	M	23	Definite							Unknown	Day		
50	F	46	Definitie-PLUS		yes		Sinus rhythm		Tall p waves	Unknown	Day	Hypertension for 15yrs, possible alcohol abuse	
51	M	39	Definite	Phenytoin, Carbamazepine						Prone	Night	Hx of seizure disorder,coronary atherosclerosis	
52	F	45	Definitie-PLUS	Phenytoin						Unknown	Day	Acute hemorrhagic pancreatitis, Hepatic cirrhosis, Old cerebral contusions, Blood alcohol 3092ug/ml	
53	M	45	Definitie-PLUS	Phenobarbital						Unknown	Day	Coronary atherosclerosis	
54	F	27	Definite							Supine	Day	Severe pulmonary arteriosclerosis	
55	F	17	non-SUDEP	Phenytoin, phenobarbital						Unknown	Day	Drowning	
56	M	30	Definitie-PLUS	Phenobarbital, carbamazepine						Prone	Night	Combined focal and generalized seizures	
57	F	73	Probable							Unknown	Day		
58	M	41	Definite							Unknown	Unknown		
59	F	18	Definite	Phenobarbital,diazepam	yes	57	Atrial bradycard	399		Prone	Night	Hx combined focal and generalized epilepsy	
60	M	55	Possible	Phenobarbital	yes	57	Sinus bradycardia			Unknown	Night		
61	F	43	Definite	Phenytoin, Phenobarbital						Prone	Night	Chronic paranoid schizophrenia	
62	M	6	Definite							Unknown	Day	Acute laryngotracheitis	
63	M	11	Definite							Unknown	Day	Prior vagals episodes, then seizures, then death	
64	M	47	Definite							Supine	Night	labelled as AMI (myocardium normal!) bystander CAD, known epilepsy	
65	F	53	Definite	Phenytoin						Supine	Day	Cardiac dysrhythmia	
66	M	21	Definite	Phenytoin , Phenobarbital, Valproate, Carbamazepine						Unknown	Day	Cerebral palsy with spastic dysplegia	
67	M	42	Definite-PLUS	Phenytoin						Prone	Night	Chronic seizure disorder,Chronic hypertension	
68	F	3	Definite							Unknown	Unknown	Known Dravet with SCN1A DNM. Had 6 minute seizure, then arrested, taken to ITU, significant anoxic brain injury, lifesupport	SCN1A unl
69	F	2	Definite-PLUS	Benzodiazepine, Topiramate						Unknown	Day	Known Dravet had witnessed arrest without preceding seizure. Autopsy showed anomalous coronary artery.	Unknown n
70	M	16	Definite							Unknown	Unknown	Dravet syndrome	Unknown n
71	F	17	Unknown	Topiramate	yes	106	Sinus arrhythm	390	Normal ECG	Unknown	Unknown	Aicardi Syndrome, outside sudden death. No autopsy available	
72	M	8	Unknown							Unknown	Unknown	Dravet syndrome	SCNA1 p(A
73	M	2	Definite							Unknown	Unknown	Dravet syndrome, Developmental delay, SCN1A Arg101Gln mutation	SCN1A p(A
74	F	68	Definitie-PLUS							Unknown	Day	Hodgkin's lymphoma, nodular sclerosing type	
75	F	56	Definite	Phenytoin, Phenobarbital						Unknown	Day	Hyperplastic marrow	
76	M	38	Definite	Phenytoin, Phenobarbital						Unknown	Night	Hx GTCS, gross picture of suffocation at death	
77	M	79	Probable							Unknown	Unknown		
78	F	33	Probable							Unknown	Unknown		
79	F	15	Probable							Unknown	Unknown		
80	M	47	Definite-PLUS	Phenytoin	yes	92	Sinus rhythm	460	Left atrial enlargement, L	Unknown	Day	Pulmonary edema	
81	F	53	Possible	Phenytoin, Phenobarbital						Unknown	Unknown		
82	M	46	Definite	Phenytoin, Phenobarbital						Unknown	Unknown	history of chronic alcoholism with chronic anxiety and inadequate personality, frontal lobotomy, convulsive disorder	
83	M	65	Definite-PLUS		yes					Supine	Day	history of alcoholism ,	
84	M	48	Possible		yes	80	Sinus rhythm	360	Normal ECG	Unknown	Unknown		
85	M	46	Definite	Phenytoin, Phenobarbital	yes					Prone	Day		
86	F	32	Definite-PLUS	Levitracetam						Unknown	Night		
87	M	56	Definite	Phenytoin						Unknown	Night	Severe OSA and obesity too, prior PE (non on autopsy)	
88	M	53	Definite-PLUS							Supine	Night		
89	M	46	Definite-PLUS	Valproate						Prone	Day	long Hx GTCS, well-controlled, sudden death, bystander CAD	
90	M	74	Definite-PLUS							Unknown	Day	idiopathic GTCS, bystander CAD and non-specific fibrosis ? Old myocarditis	
91	F	35	Definite	Carbamazepine, Valproate						Unknown	Day	Idiopathic GTCS	
92	M	36	Definite	Lamotrigine						Unknown	Unknown	post-traumatic epilepsy	
93	M	6	Definite	Levetriracetam, Topiramate						Unknown	Day	Microcephaly, probable under;lying genetic disorder. Sleep apnea	
94	F	62	Definite-PLUS	Carbamazepine						Prone	Day	LVH, bystander CAD and patchy fibrosis	
95	M	23	Definite	Valproate						Prone	Day		
96	F	50	Definite							Prone	Day	EtOH precipitaed/related seizures, sudden death, no EtOH in blood	
97	F	64	Possible							Unknown	Day	EtOH precipitaed/related seizures, sudden death	
98	M	19	Definite	Carbamazepine, Phenytoin						Unknown	Day	Niemann-pick disease	
99	F	26	Definite	Phenytoin	yes	60	Sinus rhythm	480	Complete left bundle bran	Unknown	Day		
100	M	23	Definite	Carbamazepine						Supine	Day	Tuberous sclerosis	
101	F	84	Probable	Phenytoin						Unknown	Night		
